# In vitro effect of triamcinolone and platelet-rich plasma on cytokine levels of elbow lateral epicondylitis-derived cells

**DOI:** 10.1186/s13018-022-02990-0

**Published:** 2022-02-15

**Authors:** Márcio Eduardo de Melo Viveiros, Magda Massae Hata Viveiros, Márcia Guimarães da Silva, Cláudia Aparecida Rainho, Silvana Artioli Schellini

**Affiliations:** 1 Department of Orthopedics and Traumatology, School of Medicine of Botucatu, São Paulo State University – UNESP, Botucatu, SP Brazil; 2 Department of Ophthalmology, School of Medicine of Botucatu, São Paulo State University – UNESP, Botucatu, SP 18618-970 Brazil; 3 Department of Pathology, School of Medicine of Botucatu, São Paulo State University – UNESP, Botucatu, SP Brazil; 4 Department of Chemical and Biological Sciences, Institute of Biosciences of Botucatu, São Paulo State University – UNESP, Botucatu, SP Brazil

**Keywords:** Lateral elbow epicondylitis, Triamcinolone, PRP, Cytokines, Inflammation

## Abstract

**Background:**

The pathogenesis and treatment of lateral elbow epicondylitis (LEE) are still controversial. The purpose of the current study was to evaluate the production of inflammatory cytokines by LEE-derived cells and to compare the anti-inflammatory effect of triamcinolone acetonide with platelet-rich plasma (PRP) on cytokines production in primary culture of these cells.

**Methods:**

Third passage cells from primary cultures of LEE were assessed for the production of the cytokines IL-1β, IL-6, IL-8, IL-10 and TNF-α by immune-enzymatic assay (ELISA), after the treatment with 1, 10 and 100 μM triamcinolone compared to no treated controls at the time points 6, 12, 18, 24, 48, 72 and 96 h, and to PRP at 48, 72 and 96 h.

**Results:**

The cytokines IL-6 and IL-8 were produced in high concentrations by LEE cells. One, 10 and 100 μM triamcinolone induced significant decrease in the production of IL-6 and IL-8 at 48, 72 and 96 h, adding the time point 12 h for IL-8. Compared to controls, PRP caused a significant increase in the production of IL-6 and IL-8 and there was a significant increase in IL-10 production with the use of 100 μM triamcinolone at 48 h. The production of IL1-β and TNF-α was very low and did not change when the cultures were treated with triamcinolone or PRP.

**Conclusion:**

LEE-derived cells produce IL-6 and IL-8, confirming the inflammatory nature of this condition. While triamcinolone inhibited the production of IL-6 and IL-8 by LEE cells, PRP induced an increase in these cytokines compared with controls.

**Supplementary Information:**

The online version contains supplementary material available at 10.1186/s13018-022-02990-0.

## Introduction

Tennis elbow or lateral elbow epicondylitis (LEE) is a common musculoskeletal condition that affects 1% to 3% of the adult population [1, 2]. Men and women are equally affected, occurring in productive age groups, between 35 and 50 years. As it is a debilitating condition, associated with high morbidity, often leading to prolonged absence from work, the costs associated with LEE are enormous, reflecting on the loss of productivity at work and with health care costs [3].

The pathogenesis of LEE is still controversial. Currently, it has even been suggested that LEE is a multi-factorial process with specific alterations of the psychosocial sphere [4]. Although LEE was initially considered a tendinitis, caused by tendon inflammation [5, 6], LEE had its nomenclature changed to angio-fibroblastic tendinosis, since the histopathologic examination showed that the lesion is characterized by the presence of a dense population of fibroblasts, vascular hyperplasia, and disorganized collagen with few inflammatory cells [7–9].

The mechanical stress on the tendon with a constant frequency, intensity and duration leads to morphological and molecular changes in the fibrous connective tissue, releasing pro-inflammatory cytokines produced by several cell types, such as tendon fibroblasts or even chondrocytes. These cytokines activate the intracellular signaling pathways for the release of inflammatory mediators, which will act on the degradation and degeneration of the tendon matrix [9–11]. Therefore, this continuous degeneration may result from the action of the inflammatory cytokines mediated by the fibroblasts from the injured connective tissue itself, even in the absence of inflammatory cells at the injury site [12–15].

Among the inflammatory cytokines, the interleukins IL-1β, IL-6, IL-8, IL-10 and TNF-α are the most studied regarding the development and progression of tendon diseases, as well as in healing and exercise response [16–18], being released by stromal and immunoregulatory tendon cells in presence of tissue damage and mechanical stress, altering local cells phenotype [17]. The persistence of chronic inflammation results in excessive and inadequate production of protein matrix, with consequent fibrosis [18].

IL-6 is produced by tenocytes and other cells as T-cells, B-cells, monocytes, fibroblasts, keratinocytes, endothelial, mesangial cells, adipocytes, some tumor cells [16] and can influence several cell processes, with multiple biological actions. IL-6 usually acts as a pro-inflammatory cytokine, involved in the positive regulation of inflammatory reactions and in the pain process. However, IL-6 can also act as an anti-inflammatory cytokine, through the activation of its soluble receptors.

IL-8 is also a potent chemokine, with a key role in neutrophil-mediated inflammation, which leads to the cartilage destruction and bone damage [16].

Nowadays, there is no defined treatment for LEE [19], with a large number of therapeutic options, such as corticosteroids injection, which is the most common conservative management, or platelet-rich plasma (PRP).

PRP is an autologous plasma preparation enriched with a higher platelet concentration than is normally contained in whole blood and its therapeutic use is based on the fact that a high concentration of platelets would be able to supply and release supraphysiological amounts of growth factors and cytokines to provide a regenerative stimulus, leading to the repair with low potential of fibrosis. The effect of almost all these growth factors present in PRP is to stimulate the collagen synthesis, angiogenesis and chemotaxis, although these two latter effects are known to be antagonistic to the desired result in the treatment of LEE and other inflammatory conditions. There are reports that a single injection of PRP is able to keep the patient without symptoms of LEE for up to one year, while the injection of corticosteroids shows the same result in only 51% of cases [20, 21]. It is assumed that this more long lasting effect of PRP would be due to the remodeling of a new tendon, which can to persist and to respond better to the mechanical trauma [22]. Therefore, the best treatment for LEE remains undefined [23].

The present study aimed to evaluate the production of inflammatory cytokines in the supernatant of LEE-derived cells in primary culture, comparing the effect of two widely used LEE treatments, the classic use of corticosteroids versus the use of PRP.

## Methods

Our Institutional Ethical Committee approved the study protocol and informed consent was obtained from all the donor patients. This was an in vitro study using cultures of LEE-derived cells, established from six tissue samples from unrelated patients, with mean age of 44.8 years (minimum of 35 years; maximum of 54 years), equally distributed according to sex. The inclusion criteria were adults with LEE diagnosed by clinical and imaging examination (simple radiography and ultrasound) in phases VI and VII according to Nirschl clinical classification of LEE [5]. Exclusion criteria were the presence of other associated orthopedic or rheumatological diseases and percutaneous or surgical treatment for LEE with less than six months. The patients were not using oral therapy of NSAID or steroids, only physiotherapy and analgesics.

The biological tissue samples were obtained during the surgical procedure to treat the condition. The same surgeon, following standard surgical technique, collected all samples.

### Cell culture

Under sterile conditions, in the laminar flow hood, the tissue samples were cut into small fragments of approximately 1mm^3^, which were collected into cell culture flasks, containing Dulbecco’s Modified Eagle Medium: Nutrient Mixture F-12 (DMEM/F12) (Gibco, Grand Island, NY, USA) supplemented with 5 mL/L of TC Minimal Eagle vitamins (Sigma-Aldrich, St. Louis, MO, USA), 0.01 U/ml recombinant human insulin (Gibco, Grand Island, NY, USA), 15 µg/mL Glutathione (Sigma-Aldrich, St. Louis, MO, USA), 100 IU/mL Penicillin, 40 µg/mL Gentamicin, 2 µg/mL Amphotericin-B (Gibco, Grand Island, NY, USA) and 20% fetal bovine serum (FBS) (Gibco, Grand Island, NY, USA). The culture flasks with the explants were kept in an incubator at 37ºC, with 5% CO_2_, in humid atmosphere.

The supplemented DMEM/F12 nutrient medium with 20% FBS was added every three days, until the cultures reach the semi-confluence, when cells were sub-cultured until the third passage.

### PRP preparation

During the surgical procedure, 8.5 mL of peripheral blood was collected by puncture of the anterior cubital vein of the LEE patients, in vacutainer tubes containing sodium citrate as anticoagulant and immediately taken for the processing of the PRP, following the previously described protocol for the obtaining of leukocyte-poor PRP [22, 24], which were frozen at − 80 °C until use.

### Cytokine study

Initially, the cell viability was evaluated using the MTT (3-(4,5-dimethylthiazolyl-2)-2,5-diphenyltetrazolium bromide) assay that was performed to identify the triamcinolone ideal non-cytotoxic concentrations to be used in the study of the inflammatory cytokines (Additional file 1: Fig. S1). The cytokine kinetics analysis was also carried out to verify the best evaluation times and triamcinolone concentrations to be used in the study (Additional file 2: Fig. S2). After these preliminary experiments, 5 × 10^3^ third passage LEE-derived cells were seeded in 96-well culture plates and maintained for 24 h in humidified incubator at 37 °C with 5% CO_2_ to allow their adherence. The triamcinolone group was exposed in triplicates to triamcinolone acetonide in concentrations of 1, 10 and 100 μM (which had led to an effective decrease in the production of cytokines in the previous cytokines kinetics study). The control group was exposed only to complete nutrient medium. The cultures were kept in humidified incubator at 37 °C with 5% CO_2_ for 6, 12, 18, 24, 48, 72 and 96 h. At times 48, 72 and 96 h, the effect of triamcinolone was compared to the PRP treatment.

The supernatant conditioned culture medium, which was in contact to the cells during each time point after exposure to triamcinolone, PRP and control group were collected and analyzed to determine the concentration of secreted inflammatory cytokines by enzyme-linked immunosorbent assay (ELISA). Commercially available ELISA kits (R&D Systems, Minneapolis, MN, USA) were used to quantify IL-1β, IL-6, IL-8, IL-10 and TNF-α levels in the culture supernatant. All assays were performed according to the manufacturer’s instructions. Samples in which cytokine levels were estimated to be below the sensitivity of the assay were set as equal to the sensitivity of the assay and those with concentrations at levels above standard curve were diluted and re-assayed. The assays readers were performed in an ELISA automatic reader (Epoch-BioTek, Winooski, VT, USA), at wavelength of 492 nm. The concentrations of cytokines in the conditioned culture medium were calculated on the standard curve obtained with different concentrations of the recombinant human cytokines of interest and tests were performed to determine the inter- and intra-assay variability.

### Statistical analysis

The results were submitted to statistical analysis using the two-way ANOVA test and Sidak multiple comparisons test, with a significance level of 5%, performed by the GraphPad Prism 8 software (GraphPad Software Inc, La Jolla, CA, USA).

## Results

Primary cell culture: After 7 days in culture, spindle cells started to migrate from explants, began to proliferate and reached confluence with an average of 21 days, when they were detached for the first passage. The cells were sub-cultured until the third passage, when they were subjected to viability assay and exposure to triamcinolone and PRP.

**Fig. 1:**
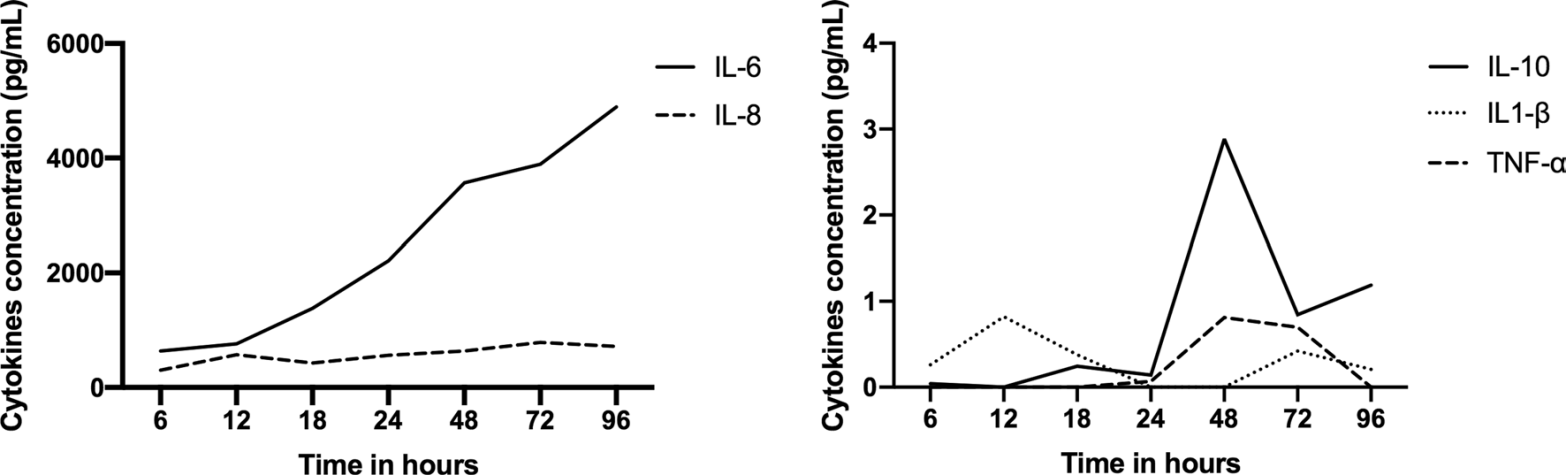
Levels of the cytokines IL-6, IL-8, IL-10, IL1-β and TNF-α in pg/mL produced by control group cultures at 6, 12, 18, 24, 48, 72 and 96 h in the study on cytokine production kinetics. Levels were determined by ELISA, without exposure to triamcinolone or PRP. *n* = 6 donors. IL, interleukin; TNF, tumor necrosis factor

Cytokine study: The cultures from control groups that were not exposed to any of the treatments showed a high production of IL-6, which quickly rose after 12 h, requiring dilution up to 1:25 for the detection of measurable IL-6 levels (Fig. 1). The minimum detectable levels in the IL-6 and IL-8 assays were, respectively, 143.29 pg/mL, 162.62 pg/mL and zero for IL-10, IL-1β and TNF-α. Intra- and inter-assay variability remained < 10.0% for all cytokines.

**Fig. 2:**
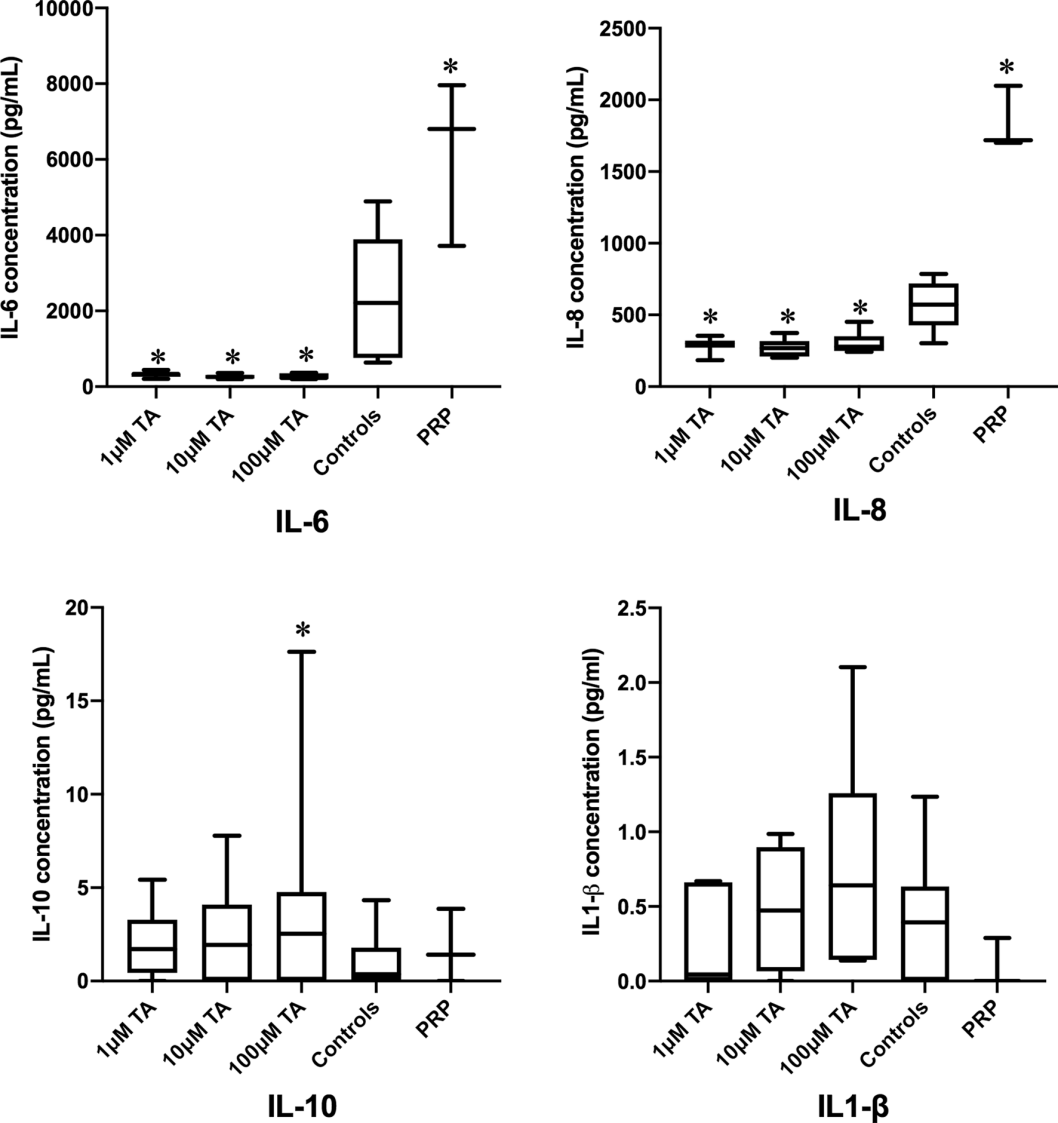
IL-6, IL-8, IL-10 and IL1-β levels in pg/mL produced by LE-derived cells exposed to 1, 10 and 100 μM triamcinolone acetonide (TA), measured at different time points, compared to PRP and controls, determined by ELISA. TA led to a significant decrease in IL-6 and IL-8 levels in all the studied concentrations, compared to controls and PRP. The production of IL-8 by the PRP group was significantly higher than the control group, at all studied time points. IL-10 levels showed a significant punctual increase with 100 μM triamcinolone at 48 h, in comparison with controls. IL1-β underwent no significant changes when the cultures were treated with triamcinolone or PRP. *n* = 6 donors. IL, interleukin. *p < 0.05 versus controls

Triamcinolone, in the three studied concentrations, led to a significant reduction in IL-6 production by LEE cells after 48 h (*p* < 0.05), 72 h (*p < 0.05 for 1 and 10 μM and **p < 0.01 for 100 μM) and 96 h (*p < 0.01), compared to controls (Fig. 2). Comparing triamcinolone to PRP, IL-6 levels were significantly lower with the use of triamcinolone in all concentrations, at 72 h (*p < 0.05) and 96 h (*p < 0.05). The cultures exposed to PRP produced IL-6 at significantly higher levels than controls at 72 and 96 h (*p < 0.05) (Fig. 2).

Regarding the production of IL-8, there was also a significant reduction with the use of triamcinolone, compared to controls at times 12 h (*p < 0.05), 48 h (*p < 0.05), 72 h (**p < 0.01 for 1, 10 and 100 μM) and 96 h (**p < 0.01 for 1 μM and (*p < 0.05 for 10 μM) (Fig. 2). Compared to PRP, the production of IL-8 by triamcinolone groups was significantly lower (*p < 0.05) than the PRP group, for all the tested concentrations, at all time points (48, 72 and 96 h). The production of IL-8 by PRP group was significantly higher (*p < 0.05) than the control group, at all studied time points (48, 72 and 96 h).

The production of IL-10 showed a significant punctual increase (*p* = 0.02) with 100 μM triamcinolone in 48 h, in comparison with unexposed controls (Fig. 2). The cytokines IL1-β and TNF-α showed no significant changes comparing to the unexposed controls when the cultures were treated with triamcinolone or PRP (Fig. 2).

## Discussion

Our results demonstrated that LEE-derived cells produced IL-6 at increasing levels, even without any external stimulus, reaching high values after 12 h. IL-8 was also produced by these cells, but at lower levels than IL-6. When exposed to triamcinolone, these cells showed a significant reduction in IL-6 and IL-8 production, compared to PRP and unexposed controls. We also observed that the PRP increased the levels of inflammatory cytokines within the entire studied period.

The present study aimed to compare the traditional treatment, based on corticosteroids, with one of the most modern treatment, using PRP. The intralesional steroid injection has the advantage of the low cost, easily acquirable and has practically no side effects [25–27], except the inherent risks related to the injection, which are the same as those for PRP.

PRP is a relatively recent treatment, with high cure rates [20, 27]. However, the response to PRP occurs only after the sixth month of application, the cost is higher than triamcinolone due to processing [20, 28] and requires the storage at ultra-lower freezers (− 80 °C), if not immediately used [29].

The current study found high IL-6 levels produced by the LEE-derived cells, possibly related to the development and progression of this condition, as it is a cytokine involved in tendinopathy in humans [30–32]. IL-8 was the second most secreted cytokine by LEE cells, a cytokine with also proven inflammatory involvement in tendinopathies [33, 34].

PRP led to an increase in IL-6 and IL-8 levels compared to controls, suggesting a pro-inflammatory effect. Thus, triamcinolone can be considered more effective than PRP in reducing inflammation in the first 96 h of treatment. The use of triamcinolone was effective in the three studied concentrations, also leading to a reduction in IL-8 levels, when compared to controls and PRP.

IL-10 showed a significant punctual increase in time 48 h with 100 μM of triamcinolone and, concomitantly, there was also an increase in IL1-β in this same evaluation time, which could justify the elevation of IL-10, since this is an anti-inflammatory cytokine, that regulates the inflammation intensity [35], which was possibly produced in order to control the rising levels of IL1-β. After this time point 48 h, IL-1-β levels gradually decreased. Although this increase in IL1-β was not statistically significant, it probably was able to stimulate the IL-10 production by LEE cells.

Despite the superior results of corticosteroids on PRP in reducing the inflammatory cytokines of LEE, they are not exempt from undesirable effects. It was already proven that triamcinolone 0.1 mg/mL led to a decrease in the viability of rotator cuff cells when exposed during 7, 14, and 21 days, due to apoptosis [36]. Therefore, the treatment with corticosteroids injection needs caution, being recommended not to use it with repetitions in short time intervals.

The PRP used in the present study was processed according to the protocol for the obtaining of leukocyte-poor PRP [24], since leukocytes, despite having important functions in tissue repair and providing protection against infectious agents, also have pro-inflammatory actions and immunological effects which can result in undesirable and opposed effects in healing, leading to an increased inflammation due to the pro-inflammatory cytokines [37, 38]. However, even this PRP preparation caused intense and significant production of IL-6 and IL-8 cytokines when compared to controls.

It is known that PRP has in its composition IL-6, IL-8, IL1-β and TNF-α [38, 39]. The development of a PRP free of pro-inflammatory factors, especially VEGF, could have better results [39]. Clinical studies comparing the use of PRP with triamcinolone confirm our results, with an initial advantage for the corticosteroid group and gradual improvement for the PRP group, with significant improvement in the PRP group only after six months [27]. However, according to the natural history of LEE, it can evolute with spontaneous improvement in six months. Another difficulty is the lack of standardization for the PRP production, which makes it difficult to compare the literature studies, as PRPs are very heterogeneous and qualitatively very different, with no strong evidence on the ideal preparation, dosage and efficacy. Given the lack of large phase II to IV trials, the effectiveness of PRP in the management of tendinopathy is not definitely proven [23, 40, 41].

Taking all these points into account, a strong point of the present in vitro study was the opportunity to work with the similar cells and submit them to the two treatments, without the influence of external factors or patient variation.

A limitation of the present study is the sample size, with cytokine measures based on primary cultures obtained from only six unrelated patients, resulting in limited cell-cycle divisions and expansion capacity. However, our primary cell cultures showed normal cell morphology and maintained many of the biological and functional features observed in vivo [42], proving that our findings are relevant.

The late effect of PRP in the treatment of LEE, which can be up to six months, also limits the assessment of the PRP effectiveness, both in in vitro and in vivo studies. A limitation of in vitro studies is the maintenance of the cells in culture after exposure to PRP that is not feasible for up to six months, since the cells will lose their phenotypic characteristics and the senescence of the cultures will invariably result in the loss of proliferation capacity, followed by apoptosis. However, the in vivo studies also have limitations, due to the possibility of spontaneous remission of LEE in most cases, not allowing to assess whether the cases that were resolved resulted from the late effect of PRP or if their resolutions resulted from the natural course of the disease.

## Conclusion

The present study shows the importance of IL-6 and IL-8 in the pathogenesis of LEE, reinforcing the inflammatory feature of this condition. In addition, our results confirm the anti-inflammatory effect of triamcinolone, the opposite to that observed with the use of PRP, which triggered an increase in the cytokines IL-6 and IL-8 LEE-derived cells in vitro.

## Supplementary Information

**Additional file 1. Figure S1** Representative quantification of the MMT assay, with the distribution of cell viability according to triamcinolone concentrations, at 24, 48, 72 and 96 hours after exposure. *p < 0.05

**Additional file 2. Figure S2** Kinetics of IL-6 and IL-8 production by LEE cells after exposure to triamcinolone, PRP and controls, determined by ELISA assay

## Data Availability

All data generated or analyzed during this study are included in this published article.

## Abbreviations

LEE: Lateral elbow epicondylitis
PRP: Platelet-rich plasma
IL-1β: Interleukin 1 beta
IL-6: Interleukin 6
IL-8: Interleukin 8
IL-10: Interleukin 10
TNF-α: Tumor necrosis factor alpha
PDGF: Platelet-derived growth factor
TGF-β: Transforming growth factor beta
VEGF: Vascular endothelial growth factor
EGF: Epidermal growth factor
bFGF: Basic fibroblast growth factor
IGF-1: Insulin-like growth factor
MTT: 3-(4,5-Dimethylthiazolyl-2)-2, 5-diphenyltetrazolium bromide)
