# Supplementary figures and images for: In vitro effect of triamcinolone and platelet-rich plasma on cytokine levels of elbow lateral epicondylitis-derived cells
Source: J Orthop Surg Res. 2022 Feb 15;17:94. doi: 10.1186/s13018-022-02990-0 (PMC8848654; doi:10.1186/s13018-022-02990-0)

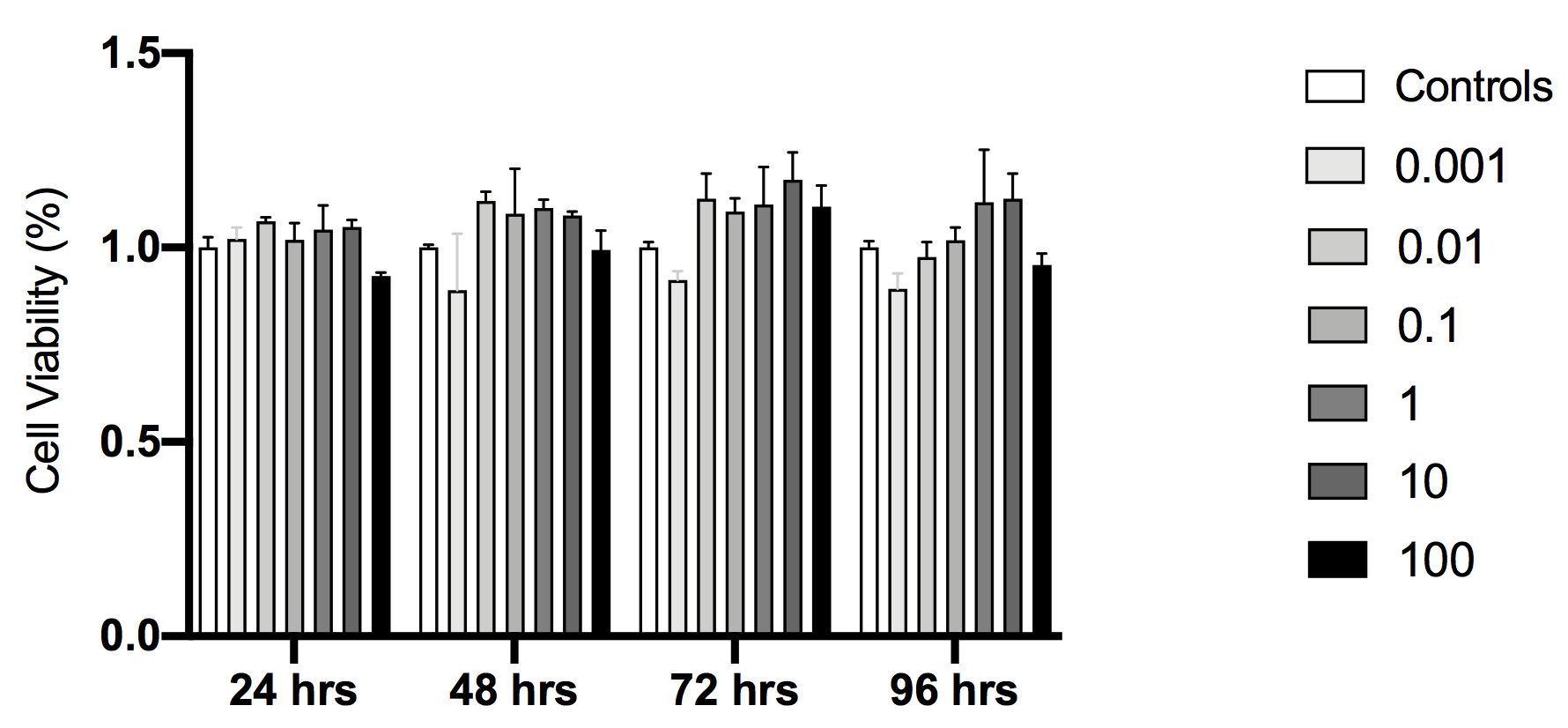

Supplement: Supplementary file 1 — Additional file 1. Figure S1 Representative quantification of the MMT assay, with the distribution of cell viability according to triamcinolone concentrations, at 24, 48, 72 and 96 hours after exposure. *p < 0.05 [file 13018_2022_2990_MOESM1_ESM.tiff]

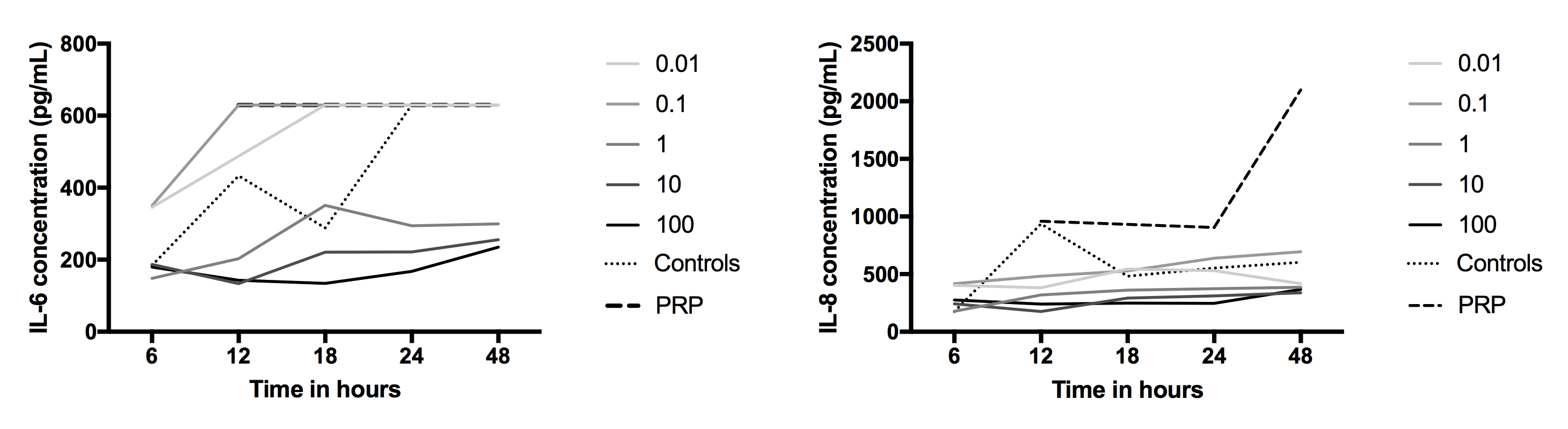

Supplement: Supplementary file 2 — Additional file 2. Figure S2 Kinetics of IL-6 and IL-8 production by LEE cells after exposure to triamcinolone, PRP and controls, determined by ELISA assay [file 13018_2022_2990_MOESM2_ESM.tiff]
